# Prevalence and distribution of selected dental anomalies among saudi children in Abha, Saudi Arabia

**DOI:** 10.4317/jced.52870

**Published:** 2016-12-01

**Authors:** Syed M. Yassin

**Affiliations:** 1 Assistant Professor, Division of Pediatric Dentistry and Orthodontics

## Abstract

**Background:**

Dental anomalies are not an unusual finding in routine dental examination. The effect of dental anomalies can lead to functional, esthetic and occlusal problems. The Purpose of the study was to determine the prevalence and distribution of selected developmental dental anomalies in Saudi children.

**Material and Methods:**

The study was based on clinical examination and Panoramic radiographs of children who visited the Pediatric dentistry clinics at King Khalid University College of Dentistry, Saudi Arabia. These patients were examined for dental anomalies in size, shape, number, structure and position. Data collected were entered and analyzed using statistical package for social sciences version.

**Results:**

Of the 1252 children (638 Boys, 614 girls) examined, 318 subjects (25.39%) presented with selected dental anomalies. The distribution by gender was 175 boys (27.42%) and 143 girls (23.28%). On intergroup comparison, number anomalies was the most common anomaly with Hypodontia (9.7%) being the most common anomaly in Saudi children, followed by hyperdontia (3.5%). The Prevalence of size anomalies were Microdontia (2.6%) and Macrodontia (1.8%). The prevalence of Shape anomalies were Talon cusp (1.4%), Taurodontism (1.4%), Fusion (0.8%).The prevalence of Positional anomalies were Ectopic eruption (2.3%) and Rotation (0.4%). The prevalence of structural anomalies were Amelogenesis imperfecta (0.3%) Dentinogenesis imperfecta (0.1%).

**Conclusions:**

A significant number of children had dental anomaly with Hypodontia being the most common anomaly and Dentinogenesis imperfecta being the rare anomaly in the study. Early detection and management of these anomalies can avoid potential orthodontic and esthetic problems in a child.

** Key words:**Dental anomalies, children, Saudi Arabia.

## Introduction

Dental anomalies are not a rare finding during routine dental examination. Developmental dental anomalies are an important category of dental symptomatology. Their incidence and degree of expression in different population groups can provide important information for phylogenic and genetic studies and help the understanding of variations within and between the different populations (1).

Developmental dental anomalies are marked deviations from the normal color, contour, size, number, and degree of development of teeth. Local as well as systemic factors may be responsible for these developmental disturbances (2). Although asymptomatic, these anomalies can lead to clinical problems, including delayed or non-eruption of the normal series of teeth; attrition; breast feeding problems; compromised esthetics; occlusal interference; accidental cusp fracture; interference with tongue space, causing difficulty in speech and mastication; temporomandibular joint pain and dysfunction; malocclusion; periodontal problems because of excessive occlusal force; post-eruptive tooth breakdown; and increased susceptibility to caries. Detailed investigation of dental anomalies is essential to prevent malocclusion, cosmetic deformities, periodontal problems, caries, and difficulties during tooth extraction and root canal treatment. In addition to clinical examinations, radiographic observations play an important role in the differential diagnoses of these anomalies (3-5). The anomalies that occur most frequently in children are missing teeth, supernumerary teeth, fused teeth and talons cusp. If anomalous is taken to mean an abnormality of the norm, then a dental anomaly is a feature of the dentition that can be expected to occur in the minority of a given population. Anomalies of the dentition hold a fascination for many dentists, more especially for those who practice Pediatric Dentistry. The presence of dental anomalies of the teeth and the likely causes may be more possibly thought provoking than features with profound consequences upon the affected dentition (6). It is vital for every practitioner to know the relative occurrence of anomalies in his/her locale in order to counsel those who may have any of these anomalies and who may seek treatment.

The aim of this study were to determine the prevalence of various dental anomalies, their distribution, differences between sexes and characteristics of selected dental anomalies in 5-12 years old Saudi children population.

## Material and Methods

This cross-sectional study was based on clinical examination and evaluation of panoramic radiographs of 1252 children (638 males and 614 females), who attended the dental clinics of Pediatric Dentistry, King Khalid University College of Dentistry after obtaining their informed consent. Only children of Saudi national aged between 5-12 years, providing consent were included in the study. The exclusion criteria of the subjects were children with history of systemic diseases, syndromes, cleft lip and or palate, tooth extracted due to caries, trauma or for orthodontic reasons, large restorations preventing observation of crown morphology, incompletely formed roots, cases of ectodermal dysplasia, Cleidocranial dysostosis and Down’s syndrome. The clinical details including the patient’s age, gender and selected anomalies were carefully checked, and recorded. A comprehensive clinical examination was carried out to detect the presence of selected dental anomalies. Digital orthopantomograms of these patients taken with orthopantomogram OP2000 (Instrumentarium) were examined in a standard manner under good lighting conditions, standardized screen brightness and resolution. The clinical and radiographic examination were studied by the principal investigator to eliminate inter examiner differences. An intra-examiner reliability test was done to calibrate the principal investigator on consistency of diagnosis for dental anomalies. The test was done by examining pictures of various dental anomalies. The scoring for each of the pictures identified correctly was recorded and repeated twice at an interval of one week. The intra-examiner reliability score for each of dental anomaly studies was high. The dental anomalies assessed were as follows:

Anomalies in Tooth Number: Congenitally missing teeth, supernumerary tooth, 

Anomalies in Tooth shape: Talons cusp, Fusion, Taurodontism,

Anomalies in Tooth Structure: Amelogenesis imperfecta, Dentinogenesis Imperfecta

Anomalies in Tooth position: Ectopic eruption, Rotation.

Anomalies such as Hypodontia and supernumerary teeth were established by clinical counting of the teeth and confirmed by radiographs. Gross deviations in tooth size that are easily discernible by clinical judgment were used to assess size anomalies. Clinically to consider a projection as a talon cusp, it must extend at least 1mm beyond the cementoenamel junction (CEJ) or half the distance from CEJ to the incisal edge (Davis and Brook 1986) (7). Fusion results in teeth with separate pulp chambers that join at the dentin level, which are determined by radiological evaluation. Taurodontism was assessed as per criteria’s laid by Schiffman and Chanannal (8). Structural anomalies were evaluated without dividing them into subgroups. The study was approved by the Research ethical committee at the college of Dentistry, King Khalid University. Data collected were entered into a spread sheet (Excel 2013; Microsoft Office) and analyzed statistically using Statistical Package for Social Sciences version 20 (SPSS Inc. Chicago, Illinois, USA). Chi square test was used for analysis. Spearman’s Rank Correlation test was performed to test the association between different groups of anomalies. For all tests, *p*- value was set at <0.05

## Results

The study comprised of 638 boys (50.9%) and 614(49.1%) girls with an age range of 5-12 years. Out of 1252 children, 284 (22.68%) had at least one dental anomaly ( Table 1). 318 children with a prevalence rate of 25.79 % had dental anomalies. The distribution by gender was 175 boys (26.95%) and 143 girls (23.28%). The distribution and prevalence of developmental dental anomalies are shown in  Table 2.

**Table 1:**
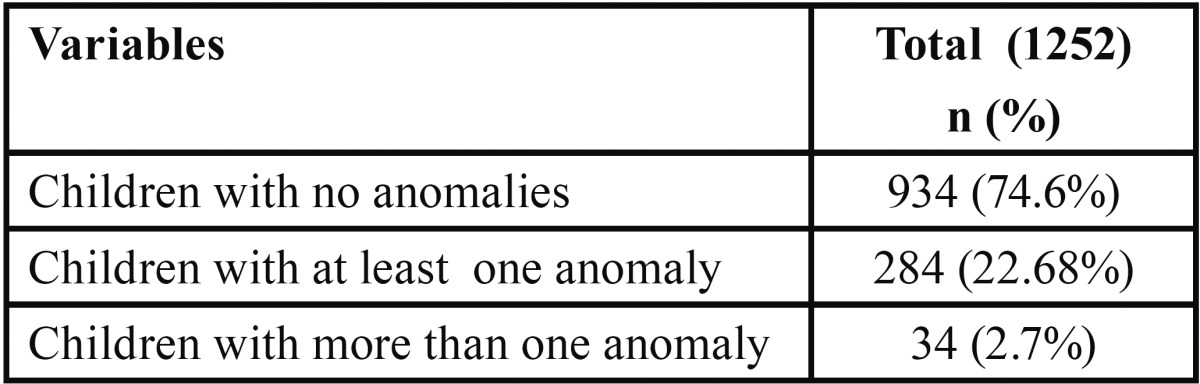
Frequencies of Dental anomalies presented in total subjects.

**Table 2:**
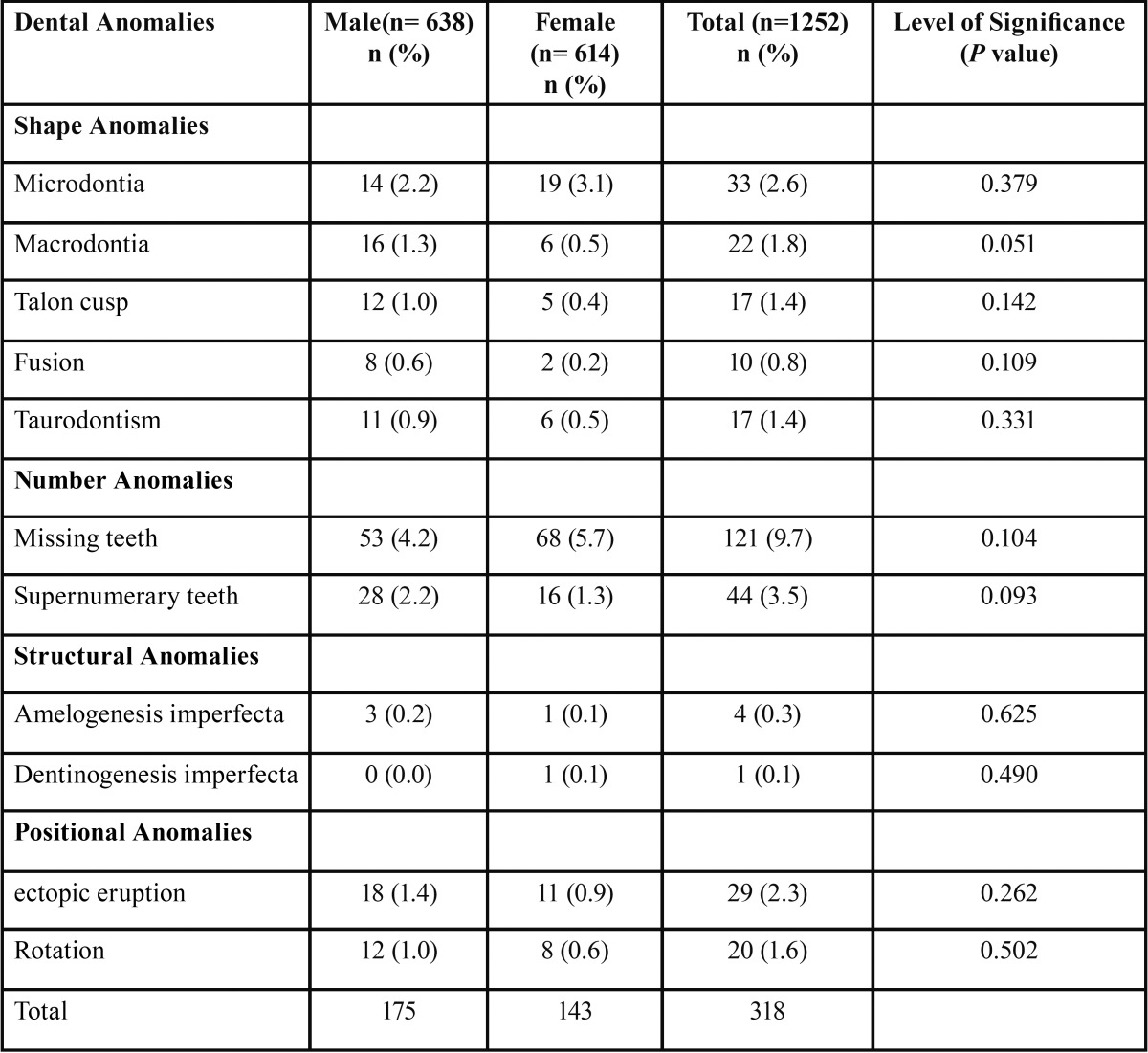
Distribution and Prevalence of Dental anomalies in 1252 children with P values from Chi square test.

Congenitally missing teeth 121(9.7%) were the most common anomaly in this study. The most commonly missing teeth were mandibular second premolars followed by maxillary permanent lateral incisors. Supernumerary tooth was the next common anomaly with the prevalence rate of 3.5%. Of the 44 supernumerary teeth, mesiodens was the most common supernumerary tooth with prevalence being more in boys (2.2%) than girls (1.3%).

Following supernumerary teeth, Microdontia was the most common anomaly, 33(2.6%) with more predominance in girls. The prevalence of other anomalies in the descending order were Ectopiceruption (2.3%), Macrodontia (1.8%), Rotation (1.6%), Taurodontism (1.4%), Talonscusp (1.4%), Fusion (0.8%), Amelogenesis Imperfecta (0.3%) and Dentinogenesis imperfecta (0.1%). When comparing all these anomalies between boys and girls, chi square test showed there were no significant differences with respect to prevalence of all dental anomalies, *P* value > 0.05 ( Table 2).

Regarding the association between different groups of anomalies, the different types of dental anomalies were correlated with each other using Spearman’s rank correlation coefficient analysis. It was found that there was significant negative correlation between Shape anomalies and Positional anomalies (Spearman’s rho *p* =0.057, *p* = 0.042). Similarly significant negative correlation was found between Number anomalies and Positional anomalies (Spearman’s rho *p* =0.079, = 0.005). There was no correlation between any other dental anomalies considered in the study ( Table 3).

**Table 3:**
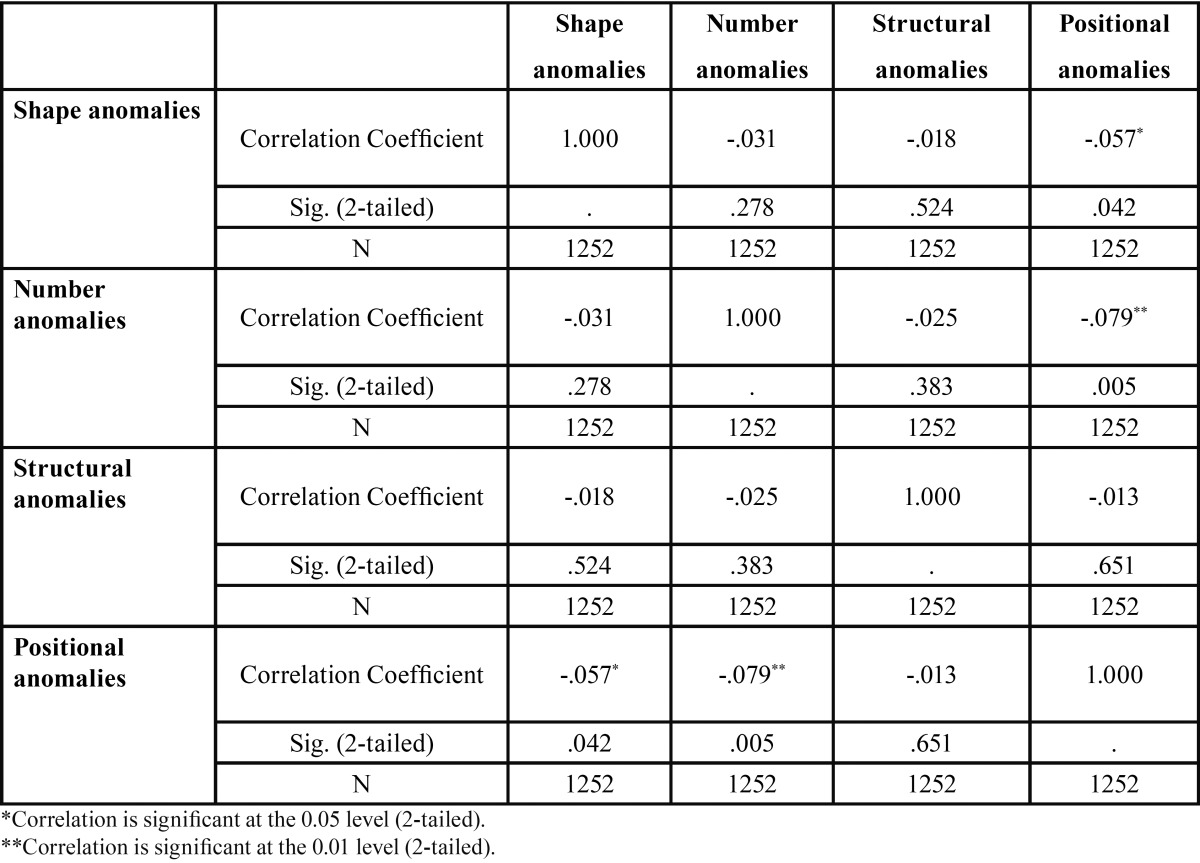
Comparative analysis between different study groups of anomalies using Spearman’s rank Correlation test.

## Discussion

The purpose of the present study was to investigate the distribution and association of dental anomalies among Saudi children. Oral health plays a crucial role in public health. Dental treatments are rather expensive health services and the combination of different modalities such as orthodontic, prosthodontic and surgical treatments can put a heavy burden on the average family’s health budget. Some frequent dental anomalies need quite expensive treatment. Treatment might be usually expensive and multidisciplinary. This highly frequent and yet expensive anomaly is of interest to numerous clinical, basic science and public health fields such as orthodontics, pediatric dentistry, prosthodontics, periodontics, maxillofacial surgery, anatomy, anthropology and even the insurance industry (9).

The results of the present study supported the findings that numerical anomalies are the most prevalent form, as similar to the one mentioned by Backman and Wahlin (10). The prevalence of Hypodontia was the most common anomaly in this study. Among the numerical anomalies congenitally missing permanent teeth were the most prevalent anomaly in children, which is similar to the findings reported by previous studies (1,2). However regarding the congenitally missing permanent teeth, the types of teeth reported to be missing varied in different ethnic groups. The European and Caucasian populations mostly reported higher missing prevalence of the mandibular second premolar followed by either the maxillary or mandibular central incisors, or the maxillary second premolars (11,12). However, the mandibular lateral incisor appears to be the most frequently missing tooth in Japanese people (13). However, excluding third molars maxillary lateral incisor was found to be the most commonly missing Permanent teeth in Indian population (1,14,15). In the present study, mandibular second premolar was the most frequently missing permanent teeth. Similar results were reported by previous study (12,13). However this is in contrast with findings byAl Emran, whoreported maxillary lateral incisors were the most frequently missing teeth in Saudi Arabian School children than mandibular second premolar (16).

Regarding congenitally missing primary teeth, frequencies above 0.5% have been reported by previous studies (17,18). In this study, four primary teeth were congenitally missing in three children with all the missing primary teeth being lateral incisors, which is similar to the finding reported by previous study (18), however this is in contrast to the results reported by Buldur *et al.*, in which central incisor were frequently missing (19). Children with Hypodontia in the primary dentition present corresponding missing permanent teeth indicating early diagnosis very essential.

In the current study, regarding gender distribution in prevalence of congenitally missing permanent teeth, 68 were seen in girls while 53 was seen in boys similar to the findings reported by previous studies (1,15,20). In the present study missing primary teeth tended to occur more in girls which are consistent with results reported by previous studies (17,18), but contrast to the findings by previous study (19), where boys had more congenitally missing primary teeth.

Zhu *et al.* (21) reported the prevalence of supernumerary teeth among the white population to be from 1% to 3% while in the Turkish population, the total percentage was 0.36% (19). In the present study, we observed supernumerary permanent teeth in 3.5% of the children. Supernumerary permanent teeth were more common in boys than girls, as reported in earlier studies (1,15,16,22). Supernumerary teeth occur frequently in the permanent dentition, more often in the anterior region as mesiodens than any other region (23,24). In the present study, mesiodens was the most common supernumerary tooth occurring more on the left than right side of the midline supporting previous studies (20,21) which is contrast to the findings reported by Amini *et al.* (25), where mesiodens were reported to occur equally between maxillary right and left quadrant.

Fusion can range from 0.5% to 5% in prevalence based on geographic, racial or genetic factors (26). The prevalence of fusion in the present study was 0.8%, which is higher than results reported by previous studies (18,20) but lower than the results reported by Buldur *et al.* (19). However the current finding is in agreement with results reported by previous studies (26,27). Prevalence of talon cusp in permanent teeth ranges from less than 1% to 8%, and 0.4% in primary teeth, with a higher frequency in males than females. In the present study, the prevalence of talons cusp was 1.4% with more predominance in boys and in maxillary lateral incisors which is in agreement with results reported by Dash *et al.* (28).

The prevalence of Microdontia was 2.6%, thus being the third most common anomaly in the present study. Gupta *et al.* (14) reported 2.58%, Atac *et al.* (20) reported 1.58%, Patil *et al.* (1) stated 1%, Kathariya *et al.* (15) reported 4.3% and Buldur *et al.* (19) reported as low as 0.3%. The reason for variation between their results might be attributed to the diagnostic criteria used for identifying and classifying dental anomalies, genetic, and racial factors. Furthermore, the types of anomalies evaluated by those studies might be another reason for this inconsistency since previous studies investigated only a few types of anomalies, not all of them.

Regarding the positional anomalies, the prevalence of ectopic eruption has been reported to be in the range of 0.7% to 7.9% (1,14). In the present study, the prevalence of ectopic eruption was found to be 2.3% which was in accordance with the finding reported by Mucerdo *et al.* (29). But contrary to that of Afify *et al.* (30), who reported ectopic eruption as low as 0.7% in Saudi population. In this study the most common ectopically erupted tooth was permanent maxillary canine which was similar to the results reported by Gupta *et al.* (14) but contrary to Afify *et al.* (30) who reported third molars and mandibular premolars to be more ectopically erupting tooth.

Rotation is another positional anomaly most often occurring because of multifactorial etiology that includes both pre eruptive and post eruptive disturbances. Results varied widely in prevalence of rotated teeth. In the present study 1.6% of the patients showed rotation with maxillary first premolars being more common. The prevalence of rotation was found to be 10.24% by Gupta *et al.* (14) and 13.2% by Kathariya *et al.* (15). This inconsistency in results might be attributed to variations in sample nature, subject ethnicity, sample size, settings of these studies as well as the accuracy of the methods and the diagnostic criteria that were used.

The least prevalent anomaly was the structural anomaly with Dentinogenesis imperfecta being the least followed by Amelogenesis imperfecta. The prevalence rate of Amelogenesis imperfecta was 0.3% while only one case of Dentinogenesis imperfecta was seen in the study, which is in line with previous results reported by Gupta *et al.* (14) in Indian population. However these results are in contrast to the results reported by Temilola *et al.* (3), in which structural anomaly was the most common form of dental anomalies with a prevalence rate of 16.1% in Nigerian population.

The study concluded that a significant number of patients (23.08%) had at least one dental anomaly. Anomalies in tooth numbers were the most common anomaly observed in the study. Congenital missing teeth was the most common anomaly seen, followed by supernumerary tooth. Structural anomalies were the least common anomaly with Dentinogenesis imperfecta being the rarest anomaly followed by Amelogenesis imperfecta. Thus to conclude, the tooth number anomalies were more common followed by shape, positional and structural anomalies respectively in Saudi children. Early recognition and management of dental anomalies can prevent child suffering from esthetic, orthodontic and periodontal problems.
